# Late-Onset Pompe Disease with Nemaline Bodies

**DOI:** 10.1155/2018/4127213

**Published:** 2018-09-27

**Authors:** E. Frezza, C. Terracciano, M. Giacanelli, E. Rastelli, G. Greco, R. Massa

**Affiliations:** Neuromuscular Diseases Unit, Department of Systems Medicine, University of Rome Tor Vergata, Rome, Italy

## Abstract

Pompe disease is an autosomal recessive disorder characterized by deficiency of alpha-glucosidase, a lysosomal enzyme, which can lead to glycogen accumulation in skeletal muscle, heart, and nervous system. Clinical presentation is highly variable, with infantile and late-onset (LOPED) forms. Although muscle biopsy findings are rather stereotyped, atypical features have been described. A 52-year-old man without a family history of muscle disorders presented with slowly progressing upper and lower limb girdle weakness and hyperCKemia. At needle EMG, a diffuse neurogenic pattern was detected. Muscle biopsy showed a selective type 1 fiber atrophy with vacuoles of various sizes, filled with PAS and acid phosphatase positive material, confirmed to be glycogen by electron microscopy (EM). Many atrophic fibers contained foci of myofibrillar material recognized as nemaline bodies (NBs) at EM. Low level of alpha-glucosidase activity in blood and molecular genetic testing confirmed the diagnosis of late-onset Pompe disease (LOPED). Major causes of hereditary and acquired NB myopathy were ruled out. In conclusion, NBs represent a novel histological finding in LOPED and characterize the atypical presentation of our case.

## 1. Introduction

Pompe disease is an autosomal recessive genetic disorder characterized by deficiency of the enzyme alpha-glucosidase. Onset and phenotypic spectrum of Pompe disease are wide and vary from the early infantile onset (IOPD) (0-1 year) to the late onset form that may present with different phenotypes, ranging from a symptomless to a severe form (LOPED) [1].

LOPED clinical spectrum is heterogeneous and includes fatigue, exercise intolerance, obstructive sleep apnea syndrome (OSAS), axial and proximal muscular weakness, and restrictive respiratory failure.

Muscle biopsy findings of LOPED differ considerably between patients and range from normal to highly abnormal. They usually comprise a vacuolar myopathy with abnormal glycogen accumulation. Vacuolization may affect preferentially either type 1 or type 2 fibers. Other findings are neurogenic-like pattern and the presence of necrotic fibers.

To confirm the suspicion of Pompe disease the quantitation of alpha-glucosidase activity is mandatory and, in case of positivity, molecular genetic testing provides finalization of the diagnosis.

## 2. Case Report

A 52-year-old man, without a history of neurological or muscle disorders, presented with slowly progressing upper and lower limb girdle weakness lasting for about 7-8 years. In particular, he complained of difficulties in going up- and downstairs and in carrying weights. He also complained of dyspnea, even with mild efforts. No dysphagia or dysphonia was reported. A recent check-up blood test showed mild hyperCKemia (CK= 468 UI/L; n. v. 10-167 UI/L).

At neurological examination he presented lumbar hyperlordosis, abdominal breath, and waddling gait. Manual muscle test (MRC) revealed bilateral and symmetric weakness of* deltoid *(4, R+L),* pectoralis* (3, R+L),* biceps b.* (4, R+L),* triceps b.* (4, R+L),* ileo-psoas* (4, R+L), and* quadriceps* (4, R+L). All remaining muscles had normal strength. Hypotrophy was evident in the axial musculature, with the presence of winged scapulae.

On blood tests, CK was slightly elevated and serum protein electrophoresis was normal.

Functional respiratory test showed a moderate restrictive ventilatory deficit.

Nerve conduction studies were unremarkable. By concentric needle EMG, abundant fibrillation potentials and positive sharp waves, associated with sporadic fasciculation potentials and complex repetitive discharges with MUPs of increased amplitude, duration, and polyphasic aspect, were detected in* tibialis anterior* muscles. Other muscles (L* deltoid* and R* vastus*) showed milder signs of neurogenic MUPs remodeling. Only in R* infraspinatus*, MUPs of reduced amplitude and duration were found, indicating a myopathic pattern.

**Figure 1:**
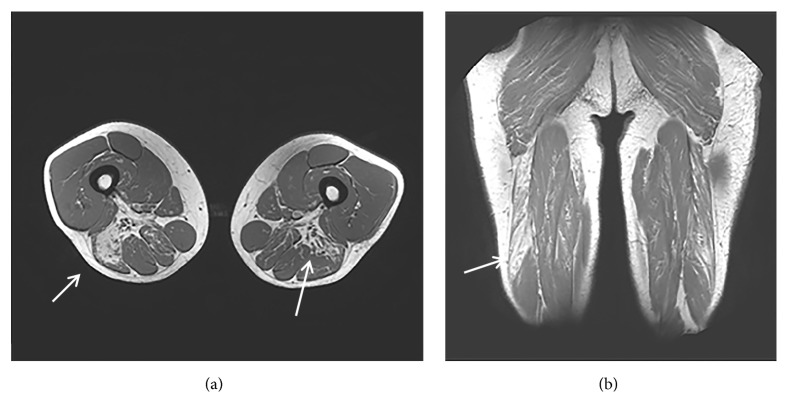
MRI of the thighs in axial T2 (a) and coronal T2 (b) sections shows bilateral fibroadipose degeneration of the* adductor* and* biceps femoris* muscles.

MRI of the thighs showed a bilateral fibro-adipose degeneration of the* adductor* and* biceps femoris* muscles, together with hypertrophy of the* gracilis *muscles (Figure 1).

**Figure 2:**
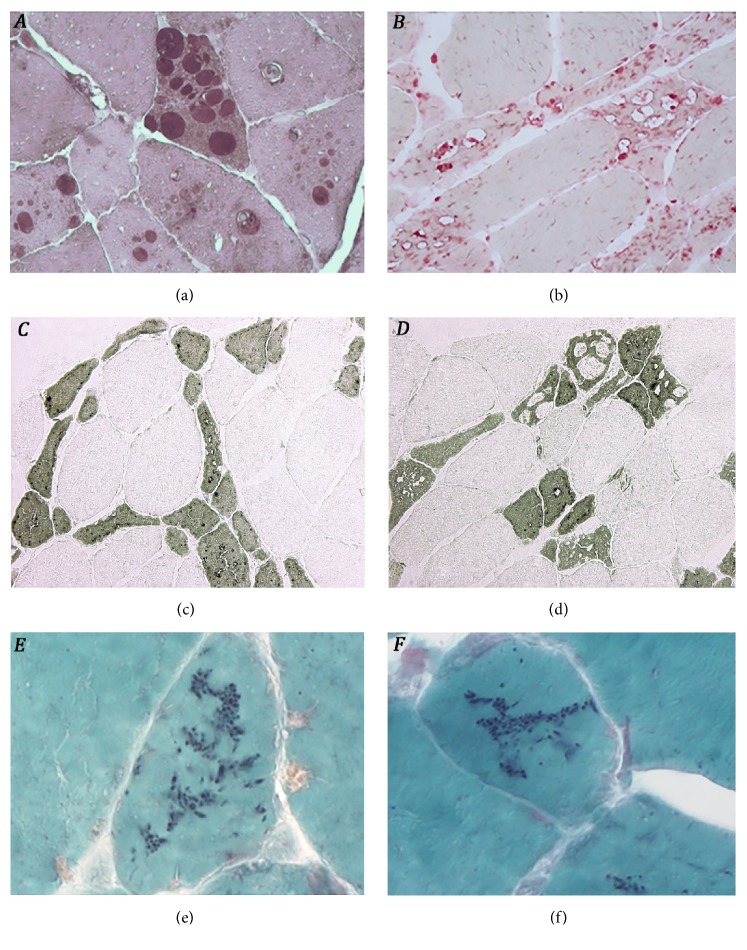
Light microscopy of the* left vastus lateralis* ((a), resin; (b)-(f), cryostat). (a) Vacuoles filled with polysaccharide material (PAS, x 40); (b) vacuoles stained positively for acid phosphatase reaction (x 20); (c) ATPase histochemistry at pH 4.3 revealed selective atrophy of type 1 fibers (x 40); (d) vacuoles are present predominantly in type 1 fibers (x 40); (e)-(f) atrophic fibers containing numerous nemaline bodies (Gomori trichrome, x 100).

**Figure 3:**
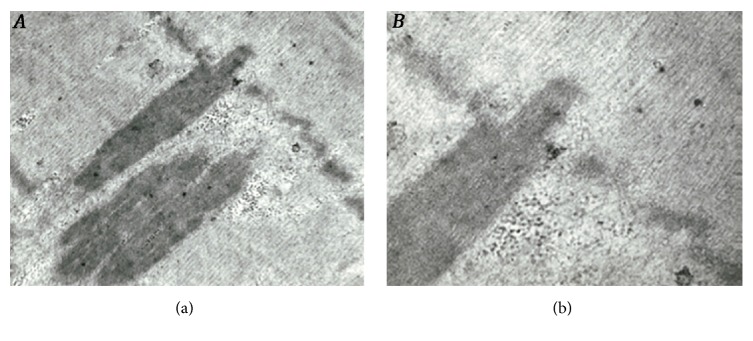
By EM, nemaline bodies are oriented consistently with the longitudinal axis of the fibers and they are composed of myofibrillar material in apparent continuity with Z-bands ((a), x 28.000; (b), x 45.000).

The patient underwent a muscle biopsy of the left* vastus lateralis* that showed few necrotic and numerous atrophic fibers, the majority of which containing small, medium, and large vacuoles. At the PAS reaction, performed on cryostat and, for a better resolution, resin sections [2], these vacuoles appeared filled with polysaccharide material that showed glycogen structure at electron microscopy (EM) observation. By the acid phosphatase reaction, all optically visible vacuoles, plus a large number of small, intracytoplasmic foci, stained positively, indicating a lysosomal nature of the vacuoles. ATPase histochemistry revealed that vacuoles were present almost exclusively in type 1 fibers, which were also diffusely atrophic, as opposed to the normal appearance of type 2 fibers. Furthermore, many of these atrophic fibers (7% of all fibers contained in transverse sections) displayed numerous, discrete, deposits of basophilic material, located centrally within the fiber cytoplasm or, in some instances, in a subsarcolemmal position (Figure 2). By EM, this material was distributed in multiple, cigar-shaped, stereotyped formations oriented consistently with the longitudinal axis of the fibers and representing nemaline bodies (NBs). These were composed of myofibrillar material in apparent continuity with Z-bands (Figure 3). No cores, or other typical alterations seen in congenital myopathies, were observed.

In order to substantiate a diagnosis of Pompe disease, we performed alpha-glucosidase (GAA) assay by mass spectrometry on dry blood spots which showed GAA 0.27 *μ*mol/L/h (n.v. 1.86–21.9 *μ*mol/L/h); alpha-galactosidase, as control enzyme, was within normal limits (8.13 *μ*mol/L/h; n.v. 5.71–49.02 *μ*mol/L/h). Consistently, GAA value in blood lymphocytes was also very low (1 nM/mg/h; n.v. 13-32 nM/mg/h). Enzymatic assay on muscle biopsy was not performed due to shortage of tissue.

Diagnosis was finally confirmed by molecular analysis, which showed a compound heterozygosis for the known c.-32-13 T>G splice mutation and the known c.2544delC deletion (p.Lys849fs) in the* GAA* gene.

## 3. Discussion

The clinical presentation of LOPED spans a wide range of severity, from asymptomatic to seriously impaired patients. In our case, the degree of clinical involvement could be placed in the middle of this range, with a muscle distribution typical of LOPED.

EMG findings are usually suggestive of a myopathy, although neurogenic changes were described in some cases. The almost “pure” neurogenic features of our patient may represent a less frequent EMG pattern, possibly indicating secondary neurogenic modifications induced by a chronic myopathic process.

Classic LOPED histopathology shows a vacuolar myopathy with abnormal glycogen accumulation, which may affect preferentially either type 1 or type 2 fibers, and increased staining for acid phosphatase activity. Atypical features have been reported as the presence of reducing bodies-like globular inclusions, lobulated fibers, COX-negative fibers, and ragged-red fibers [3].

In addition to the common histopathological alterations, in the present case we found abundant NBs grouped in the cytoplasm of a high percentage of muscle fibers, mainly of type 1.

The coexistence of glycogen-filled vacuoles, NB, and fiber atrophy with an overwhelming prevalence among type 1 fibers indicates that, for unknown reasons, this fiber type is selectively affected in the present case. To our knowledge, this is the first report of NB in Pompe disease.

Nemaline bodies, or rods, contain Z-line material and thin filament material (alpha-actinin, actin and tropomyosin with or without desmin at the periphery). They can be visualized on light microscopy by the Gomori trichrome stain, appearing as dark blue structures localized in the sarcoplasm, predominantly in regions with disrupted sarcomere structure.

NBs are characteristic histological findings in nemaline body myopathy, a congenital myopathy determined by mutations in different genes (Table 1). They are usually seen in both type 1 and type 2 muscle fibers, except in patients with* TPM3 *mutations, where they are limited to type 1 fibers [4].

However, NBs have been described as nonspecific alterations in many different conditions, either acquired or genetically determined (Table 1), and they probably represent a common response of muscle fibers to a given pathologic situation. The term Sporadic Late Onset Nemaline Myopathy (SLONM) has been recently proposed to describe patients with a late-onset myopathy, no family history, and NB as the main histological feature [5]. It includes different etiologies among which the most common is monoclonal gammopathy of undetermined significance (MGUS) [6]. Myopathy related to HIV, idiopathic inflammatory myopathy, and neurogenic conditions such as ALS, diabetic polyneuropathy, and vasculitis have also been associated with NB [7]. As to glycogen storage disease, NBs have been described only in one case of polyglucosan body myopathy with mutation in the Glycogenin-1 gene [8].

In our case, the most common causes of NB myopathy, either hereditary or acquired, were ruled out by molecular-genetic testing of* ACTA1* and* NEB* genes, screening for HIV infection and MGUS. Nonetheless, at present we cannot rule out with certainty the possibility of mutations in genes less frequently involved in NB myopathy (i.e.,* TPM3*) that could produce a “double-trouble” effect, at least on the genetic and histopathological ground. However, the sporadic and late-onset presentation of the disease and the clinical phenotype lacking dysmorphisms in our patient are not suggestive of those entities.

In conclusion, NBs represent a novel histological finding in GAA deficiency myopathy. We believe that the observation of diffused NB in an otherwise nondiagnostic biopsy from an adult myopathic patient may suggest including LOPED in a panel of possible etiologies.

## Figures and Tables

**Table 1:** Genetic and acquired etiologies of NB myopathy.

**Etiologies of NB Myopathy**	**NB in other neuromuscular disorders**
**AD: ** *NEB, ACTA1, TPM3, TPM2*	**Myopathy**
**AR: ** *ACTA1, TPM3, TPM2, TNNT1, CFL2, KBTBD13, KLHL40, KLHL41, LMOD3, MYPN, MYO18B*	Idiopathic inflammatory myopathies
	Acute alcoholic myopathy
	Myotonic dystrophy
	Sarcoglycanopathies
	Mitochondrial myopathy
	*GYG1* polyglucosan body myopathy
	Late-onset Pompe disease
**Acquired**	**Neuropathy**
MGUS	Spinal muscular atrophy
HIV- associated myopathy	Amyotrophic lateral sclerosis
	Charcot-Marie-Tooth disease
	**Other**
	Hypothyroidism
	Chronic renal failure

**Keys:** *genes* are written in *italic* font; AD, autosomal dominant; AR, autosomal recessive; NB, nemaline body; MGUS, monoclonal gammopathy of undetermined significance.
